# Etiologies and predictors of mortality in an all-comer population of patients with non-ischemic heart failure

**DOI:** 10.1007/s00392-023-02354-6

**Published:** 2024-01-15

**Authors:** S. Göbel, A. S. Braun, O. Hahad, U. von Henning, M. Brandt, K. Keller, M. M. Gaida, T. Gori, H. P. Schultheiss, F. Escher, T. Münzel, P. Wenzel

**Affiliations:** 1 Cardiology I – Department of Cardiology, University Medical Center Mainz (Johannes Gutenberg University Mainz), Langenbeckstr. 1, 55131 Mainz, Germany; 2 German Center for Cardiovascular Research (DZHK), Partner Site Rhine Main, Mainz, Germany; 3 Center for Thrombosis and Hemostasis (CTH), University Medical Center Mainz (Johannes Gutenberg-University Mainz), Mainz, Germany; 4 Institute of Pathology, University Medical Center Mainz (Johannes Gutenberg University Mainz), Mainz, Germany; 5 TRON, Translational Oncology at the University Medical Center Mainz, Mainz, Germany; 6 Institute of Cardiac Diagnostics and Therapy (IKDT), Berlin, Germany; 7 Department of Cardiology, Angiology and Intensive Care Medicine, Deutsches Herzzentrum der Charité, Campus Virchow Klinikum, Berlin, Germany; 8 German Center for Cardiovascular Research (DZHK), Partner Site Berlin, Berlin, Germany

**Keywords:** Heart failure, Endomyocardial biopsy, All-cause mortality, Cardiac amyloidosis

## Abstract

**Background:**

Despite progress in diagnosis and therapy of heart failure (HF), etiology and risk stratification remain elusive in many patients.

**Methods:**

The My Biopsy HF Study (German clinical trials register number: DRKS22178) is a retrospective monocentric study investigating an all-comer population of patients with unexplained HF based on a thorough workup including endomyocardial biopsy (EMB).

**Results:**

655 patients (70.9% men, median age 55 [45/66] years) with non-ischemic, non-valvular HF were included in the analyses. 489 patients were diagnosed with HF with reduced ejection fraction (HFrEF), 52 patients with HF with mildly reduced ejection fraction (HFmrEF) and 114 patients with HF with preserved ejection fraction (HFpEF). After a median follow-up of 4.6 (2.5/6.6) years, 94 deaths were enumerated (HFrEF: 68; HFmrEF: 8; HFpEF: 18), equating to mortality rates of 3.3% and 11.6% for patients with HFrEF, 7.7% and 15.4% for patients with HFmrEF and 5.3% and 11.4% for patients with HFpEF after 1 and 5 years, respectively. In EMB, we detected a variety of putative etiologies of HF, including incidental cardiac amyloidosis (CA, 5.8%). In multivariate logistic regression analysis adjusting for age, sex and comorbidities only CA, age and NYHA functional class III + IV remained independently associated with all-cause mortality (CA: HR_perui_ 3.13, 95% CI 1.5–6.51; *p* = 0.002).

**Conclusions:**

In an all-comer population of patients presenting with HF of unknown etiology, incidental finding of CA stands out to be independently associated with all-cause mortality. Our findings suggest that prospective trials would be helpful to test the added value of a systematic and holistic work-up of HF of unknown etiology.

**Graphical abstract:**

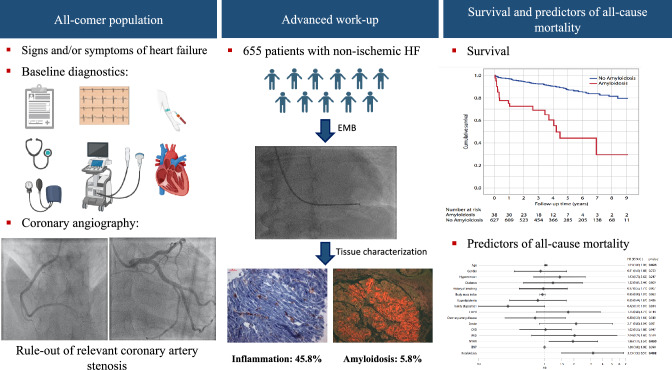

**Supplementary Information:**

The online version contains supplementary material available at 10.1007/s00392-023-02354-6.

## Introduction

Coronary artery disease and arterial hypertension are the predominant risk factors for the development of heart failure (HF) [1]. In cases of non-ischemic and non-valvular HF, the underlying etiology is likely to be multifactorial and cannot always be detected by established diagnostic procedures such as coronary angiography, echocardiography, cardiac magnetic resonance imaging (cMRI) or additive genetic testing. Considering recent advances in the development of new therapies for specific myocardial diseases, an accurate and early diagnosis is mandatory to provide the most appropriate therapy for the patient and improve survival. Endomyocardial biopsy (EMB) integrated into the diagnostic work-up may improve diagnostic accuracy of unexplained HF [2] as it enables detailed tissue characterization. A search for etiologies is recommended in current guidelines for HFpEF [3] and with less stringency also for HFrEF [4, 5]. Initially, EMB was predominantly used for monitoring of heart transplant rejection [6, 7]. EMB has been demonstrated to be a safe procedure if performed in high volume centers by experienced operators [8–11]. Until today, it remains controversial, if routine EMB helps to risk-stratify patients with HF of unknown etiology or predict their outcomes. We therefore set out to investigate the long-term follow-up in an all-comer population with HF of unknown etiology that had undergone a structured diagnostic workup including routine EMB, and identify factors that may predict outcomes.

## Methods

### Study population

The Mainz Endomyocardial Biopsy in Heart Failure Study (My Biopsy-HF Study; German Clinical Trials Register number: 22178) is a retrospective monocentric study conducted at the University Medical Center Mainz (Germany) to investigate the etiology of HF in an all-comer population of patients with non-ischemic and non-valvular HF [2]. Patients presenting with symptoms and/or signs of HF at the Heart Failure Outpatient Clinic, the emergency department or the chest pain unit of the University Medical Centre Mainz between 14/10/2012 and 01/03/2021, who underwent endomyocardial biopsy (EMB) were available for the present analysis. Decision to obtain EMB of the patients was based on current guidelines of the American Heart Association (AHA), the American College of Cardiology (ACC) and the European Society of Cardiology (ESC) in 2007 [4]. Prior to EMB, echocardiography was performed to rule out valvular heart disease, as well as a left heart catheterization to rule out ischemic heart disease. Based on the patients’ medical reports, the following data were included in the present analysis: personal history, clinical presentation, medication, laboratory results, electrocardiogram, echocardiographic findings as well as the results of EMB and genotyping. Informed consent was obtained from all included individuals to use EMB tissue samples for further scientific purpose.

### Echocardiography

A comprehensive standardized echocardiographic examination was performed by trained and certified medical staff in all patients in the echocardiographic laboratory of the University Medical Center Mainz using either a Philips ie33, a GE E9 or Siemens Acuson s2000 machine according to standard operating procedures (SOP). Routinely 2–4 cardiac cycles were obtained at frame rates of 50–100 fps and digitally transferred into a picture archiving and communication system (Xcelera) for offline analysis. All structural and functional measurements were made according to current ASE/EACVI recommendations [12].

Detailed information on the assessment of systolic and diastolic function is provided in the Supplementary Appendix.

### Endomyocardial biopsy

Endomyocardial biopsy is described in detail in the Supplementary Appendix.

#### Tissue processing, polymerase chain reaction, histology and immunohistochemistry

Information on tissue processing, histology and immunohistochemistry is provided in detail in the Supplementary Appendix.

### Genotyping

Technical aspects of genotyping are described in detail in the Supplementary Appendix.

### Outcome measures

#### Mortality

All-cause mortality was defined as the main study outcome. Follow-up was performed through March 2022 using the medical record system. In addition to death notes in clinical care, information on death/survival was also regularly obtained and updated from the mortality registry of the state of Rhineland-Palatinate (“Mortalitätsregister Rheinland Pfalz”). Information on the date of death was available, however, the underlying cause could not be provided, so that we can solely report on all-cause mortality.

### Ethics approval

The retrospective monocentric Mainz Endomyocardial Biopsy in Heart Failure Study (My Biopsy-HF Study, German Clinical Trials Register number: 22178) was approved by the Ethics Committee of Rhineland Palatinate to be in accordance with the legal regulations and the declaration of Helsinki.

### Statistical analysis

Univariate and multivariate logistic regression models were used to analyze predictors of all-cause mortality in patients with HF. Results are presented as odds ratio (OR) and 95% confidence intervals (CI). The multivariate regression models were adjusted for age and sex, classical cardiovascular risk factors (i.e. history of smoking, arterial hypertension, diabetes mellitus, hyperlipidemia, obesity, family disposition) as well as several comorbidities (chronic obstructive pulmonary disease, chronic kidney disease, coronary artery disease, stroke peripheral artery disease and amyloidosis). A probability value of *p* < 0.05 was considered statistically significant. Overall survival was estimated by the Kaplan–Meier method and tested for differences in survival between groups by the log-rank test. All statistical analyses were performed with SPSS (version 22.0; IBM Corp.).

## Results

### Sample characteristics stratified for phenotype of heart failure

The analysis sample comprised 655 patients (70.9% men) who presented with symptoms and/or signs of HF at the outpatient clinic, the emergency department or the chest pain unit of the University Medical Centre Mainz between October 2012 and March 2021. 378 (57.7%) presented in New York Heart Association (NYHA) functional class I or II and 277 (42.3%) with NYHA functional class III or IV. 489 (74.7%) patients were diagnosed with HF with reduced ejection fraction (HFrEF), 52 (7.9%) patients with HF with mildly reduced ejection fraction (HFmrEF) and 114 (17.4%) patients with HF with preserved ejection fraction (HFpEF). Patients with HFrEF were older compared to patients with HFmrEF and HFpEF (HFrEF vs. HFmrEF vs. HFpEF: 56 [47/65] vs. 53 [43/65] vs. 53 [39/67]) and were more likely to present in NYHA functional class III or IV (47.1% vs. 25% vs. 29.8%). The expression of natriuretic peptides (i.e. BNP) was highest amongst patients with HFrEF (597 [184/1266] vs.152 [35/527] vs. 159 [31/411] pg/ml), whereas the expression of high-sensitive cardiac troponin I was highest in patients with HFmrEF (21 [9/53] vs. 55 [3/224] vs. 29 [7/176] pg/ml). A detailed sample characterization including comorbidities, cardiovascular risk factors as well as functional parameters and biomarker expressions stratified for phenotypes of our cohort of non-ischemic and non-valvular HF is provided in Table 1.

**Table 1:** Baseline characteristics stratified for phenotype of heart failure

Characteristics	HFrEF *N* = 489	HFmrEF *N* = 52	HFpEF *N* = 114
Age [years]	56 (47/65)	53 (43/65)	53 (39/67)
Sex (female)—% (*n*)	29.7% (145)	34.6% (18)	24.6% (28)
BMI	27 (24/30.8)	27 (23/31.9)	26.1 (24.2/29.7)
*Cardiovascular risk factors*			
Diabetes—% (*n*)	17.6% (86)	23.1% (12)	12.3% (14)
Arterial hypertension % (*n*)	51% (249)	50% (26)	50.9% (58)
Dyslipidemia—% (*n*)	20.1% (98)	19.2% (10)	22.8% (26)
Family history of cardiovascular disease—% (*n*)	19.6% (96)	25% (13)	22.8% (26)
Smoking—% (*n*)	45.2% (221)	30.8% (16)	42.1% (48)
*Comorbidities*			
Atrial fibrillation—% (*n*)	14.5% (71)	9.6% (5)	7.1% (8)
Coronary artery disease^1^—% (*n*)	14.9% (73)	9.6% (5)	18.4% (21)
Stroke—% (*n*)	4.5% (22)	9.6% (5)	4.4% (5)
Peripheral artery disease—% (*n*)	3.1% (15)	1.9% (1)	0.9% (1)
COPD—% (*n*)	9.4% (46)	0% (0)	4.4% (5)
Chronic kidney disease—% (*n*)	14.1% (69)	7.7% (4)	22.8% (26)
*Left ventricular structure/function*			
LV ejection fraction [%]	25 (20/35)	45 (45/45)	55 (55/60)
LV end-diastolic pressure (mmHg)	18 (13/26)	16 (12/23)	19 (13/23)
Troponin I [pg/ml]	21 (9/53)	55 (3/224)	29 (7/176)
BNP [pg/ml]	597 (184/1266)	152 (35/527)	159 (31/411)
*Signs and symptoms at presentation*			
NYHA I/II—% (*n*)	52.9% (259)	75% (39)	70.2% (80)
NYHA III/IV—% (*n*)	47.1% (230)	25% (13)	29.8% (34)
Angina pectoris—% (*n*)	22.9% (112)	30.8% (16)	32.5% (37)
Oedema—% (*n*)	23.1% (113)	11.5% (6)	18.4% (21)

Data are presented as relative and absolute frequencies of subjects for binary variables and median with 1st and 3rd quartile for continuous traits

*BMI* body mass index, *COPD* chronic obstructive pulmonary disease, *BNP* brain natriuretic peptide, *LV* left ventricular, *NYHA* New York Heart Association

^1^No history of myocardial infarction, and no relevant coronary stenosis requiring revascularization revealed by invasive coronary angiography

### Etiology of heart failure based on endomyocardial biopsy findings

Based on the results of both histological and immunohistochemical analyses of endomyocardial biopsy specimens including virus detection, virus-negative cardiomyopathy was detected in 218 patients (33.3%) being the most common EMB-finding in the present analysis sample. According to the phenotype of HF, patients with evidence of virus-negative cardiac inflammation predominantly presented with HFrEF (77.5% of all patients with virus-negative cardiac inflammation). Active virus replication was detected in 10 patients with cardiac inflammation (1.5% of the whole study population). 7 patients (1.1% of the whole study population) were diagnosed with active myocarditis and 6 patients (0.9% of the whole study population) with giant cell myocarditis. Equivalent to patients with virus-negative cardiac inflammation, patients with the aforementioned types of inflammatory cardiomyopathies also presented mostly with HFrEF.

Overall, 38 patients (5.8%) had histological evidence of cardiac amyloidosis (CA). Further histological sub-specification revealed that 15 (2.3%) CA patients were diagnosed with immunoglobulin light chain cardiac amyloidosis (AL-CA) and 23 (3.5%) with transthyretin cardiac amyloidosis (ATTR-CA). Histological analysis revealed unspecific findings in overall 160 patients (24.4%). A detailed overview of all EMB findings in our patient population is depicted in Table 2, stratified for phenotype of HF.

**Table 2:** Diagnosis based on EMB findings stratified for phenotype of heart failure

Characteristics	HFrEF *N* = 489	HFmrEF *N* = 52	HFpEF *N* = 114
Unspecific finding—% (*n*)	24.3% (119)	26.9% (14)	23.7% (27)
Dilative Cardiomyopathy—% (*n*)	15.3% (75)	1.9% (1)	1.8% (2)
Hypertrophic cardiomyopathy—% (*n*)	1.2% (6)	1.9% (1)	7% (8)
Hypertensive heart disease—% (*n*)	0.6% (3)	0% (0)	0.9% (1)
ARVD—% (*n*)	0.2% (1)	1.9% (1)	0% (0)
Amyloidosis—% (*n*)	2.7% (13)	11.5% (6)	16.7% (19)
Sarcoidosis—% (*n*)	0.4% (2)	1.9% (1)	1.8% (2)
Post myocarditis—% (*n*)	9.4% (46)	11.5% (6)	6.1% (7)
Active myocarditis—% (*n*)	1% (5)	0% (0)	1.8% (2)
Erythroparvovirus without inflammation—% (*n*)	4.7% (23)	11.5% (6)	1.8% (2)
Erythroparvovirus with inflammation—% (*n*)	1.2% (6)	0% (0)	2.6% (3)
Coxsackie without inflammation—% (*n*)	0.6% (3)	1.9% (1)	0% (0)
Coxsackie with inflammation—% (*n*)	0.2% (1)	0% (0)	0% (0)
Erythroparvovirus/HHV reactivation without Inflammation—% (*n*)	1.8% (9)	5.8% (3)	1.8% (2)
Giant cell myocarditis—% (*n*)	0.8% (4)	0% (0)	1.8% (2)
Toxic cardiomyopathy—% (*n*)	0.8% (4)	0% (0)	0% (0)
Virus negative cardiac inflammation—% (*n*)	34.6% (169)	23.1% (12)	32.5% (37)

Data are presented as relative and absolute frequencies of subjects for binary variables. ARVD, arrhythmogenic right ventricular dysplasia

### Genotyping

All patients with ATTR-CA underwent additional genotyping to further analyze the underlying cause of ATTR-CA, revealing 17 patients with wild-type transthyretin amyloidosis (ATTRwt) and 6 patients with variant transthyretin amyloidosis (ATTRv). Besides patients with histological proven transthyretin amyloidosis, genotyping was also performed in 84 additional patients in whom a genetic cause was suspected following detailed counselling. Supplemental Table 1 provides an overview of the total numbers of performed genotyping stratified for EMB-based diagnosis. All detected mutations stratified for phenotype of HF are depicted in detail in Supplemental Table 2.

### Survival of patients and predictors of all-cause mortality

**Fig. 1:**
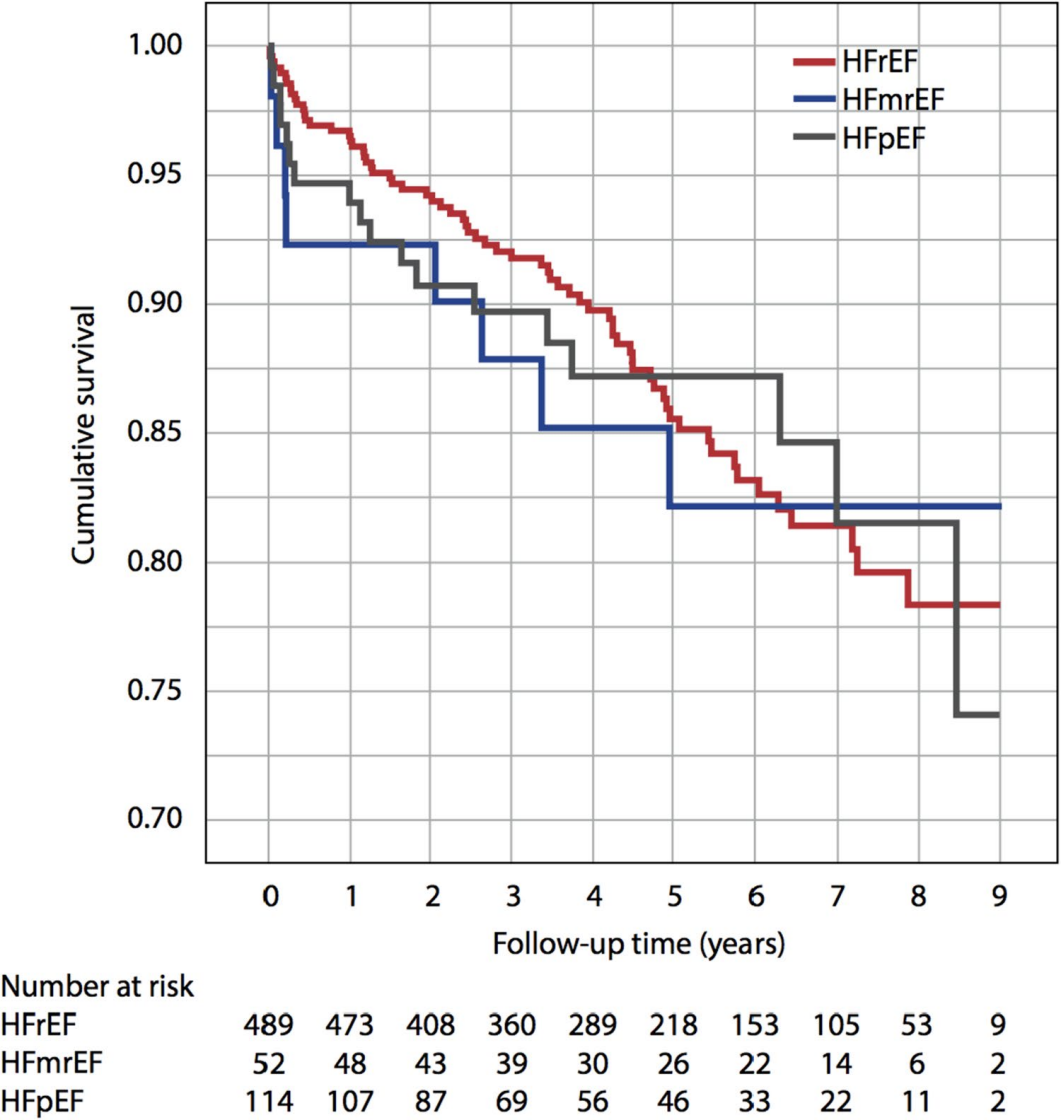
Long-term survival stratified for phenotype of heart failure. Kaplan–Meier curves indicating cumulative survival are displayed for patients with heart failure with reduced ejection fraction, heart failure with mildly reduced ejection fraction and heart failure with preserved ejection fraction

**Fig. 2:**
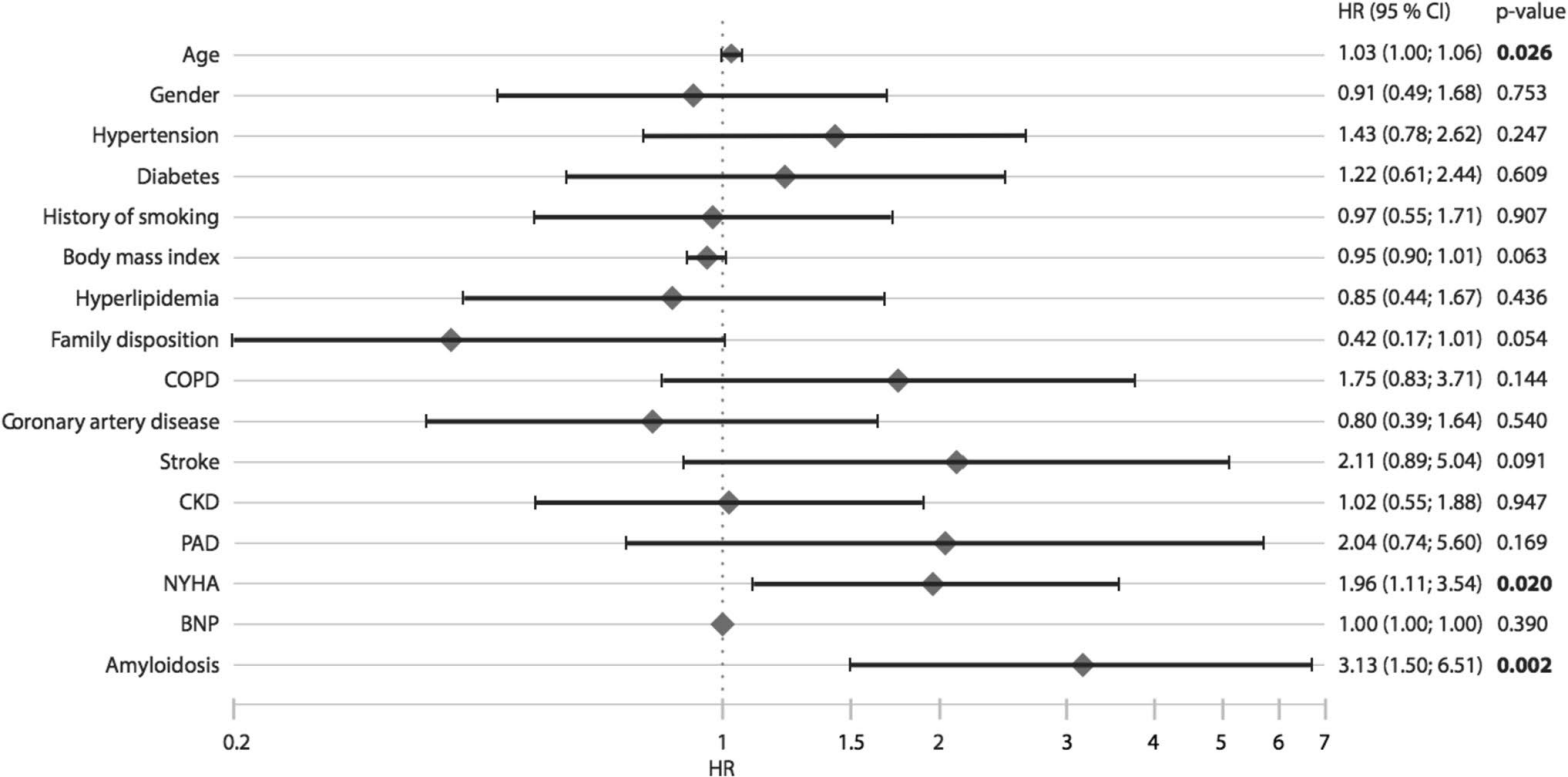
Predictors of all-cause mortality. Multivariate linear regression analysis was performed to investigate the association of cardiac amyloidosis with overall mortality, adjusting for age, sex, arterial hypertension, diabetes mellitus, history of smoking, body mass index, hyperlipidemia, family disposition, chronic obstructive pulmonary disease, coronary artery disease, stroke, chronic kidney disease, peripheral artery disease, amyloidosis and NYHA functional class. *HR* indicates hazard ratio, *CI* confidence interval, *COPD* chronic obstructive pulmonary disease, *CKD* chronic kidney disease, *PAD* peripheral artery disease, *BNP* brain natriuretic peptide, *NYHA* New York Heart Association functional class

After a median follow-up of 4.6 (2.5/6.6) years, 94 deaths were enumerated (HFrEF: 68; HFmrEF: 8; HFpEF: 18). This equated to mortality rates of 3.3% and 11.6% for patients with HFrEF, 7.7% and 15.4% for patients with HFmrEF and 5.3% and 11.4% for patients with HFpEF after 1 and 5 years, respectively (Fig. 1). The corresponding annualized event rates were 16 events/1 year and 57 events/5 years for patients with HFrEF, 4 events/1 year and 8 events/5 years for patients with HFmrEF and 6 events/1 year and 13 events/5 years for patients with HFpEF, respectively. Overall, 25 patients died out of hospital and 69 during hospitalization. All-cause mortality was significantly higher in patients presenting in NYHA functional class III or IV compared with patients presenting in NYHA functional class I or II as indicated by a hazard ratio of 2.18 (hazard ratio [HR] per unit increase [ui], 2.18, 95% CI, 1.41–3.37). After adjustment for age, classical cardiovascular risk factors and comorbidities, only CA, age and NYHA functional class were independently associated with increased all-cause mortality (CA: HR_perui_ 3.13 [1.5–6.51]; *p* = 0.002; Age: HR_perui_1.03 [1.00–1.06]; *p* = 0.026; NYHA functional class III/IV: HR_perui_ 1.96 [1.11–3.54]; *p* = 0.02) (Fig. 2). As cardiac inflammation in general as defined according to Caforio et al. [13] was not associated with all-cause mortality, we performed a subgroup analysis of patients with inflammatory cardiomyopathy (i.e. composite of active myocarditis; erythroparvovirus with inflammation; coxsackie with inflammation; giant cell myocarditis; virus-negative cardiac inflammation). Stratification of this subgroup according to markers of inflammation revealed that perforin-positive inflammation (compared with perforin-negative inflammation) was significantly associated with all-cause mortality in univariate regression analysis (HR_perui_ 2.28 95% [CI 1.04/4.95]; *p* = 0.038), as well as after adjustment for age and sex (HR_perui_ 2.29 [95% CI 1.04/5.06]; *p* = 0.04) (Supplemental Fig. 1).

### Patient characteristics and outcome stratified for the presence of amyloidosis

We next stratified the study population for the presence of CA. Patients with CA were older compared to patients without CA (70 [65/76] vs. 55 [44/64]; *p* < 0.001), had a higher prevalence of comorbidities like chronic kidney disease (44.7% vs. 13.7%; *p* < 0.001) and history of stroke (13.2% vs. 4.5%; *p* = 0.023) and showed a higher left ventricular ejection fraction based on echocardiographic examination (50% [40/55] vs. 30% [20/40]; *p* < 0.001) as well as interventricular septal thickness (1.6 cm [1.4/2.1] vs. 1.1 cm [1.0/1.5]; *p* = 0.002). A detailed sample characterization including comorbidities, cardiovascular risk factors as well as functional parameters and biomarker expressions stratified for the presence of amyloidosis, is provided in Table 3.

**Table 3:** Patient characteristics stratified for presence of cardiac amyloidosis

Characteristics	CA *N* = 38	No CA *N* = 627	*p* value
Age [years]	70 (65/76)	55 (44/64)	** < 0.001**
Sex (female)—% (*n*)	18.4% (7)	29.8% (187)	0.096
BMI	25.3 (24.1/28.7)	26.9 (24/30.9)	0.215
*Cardiovascular risk factors*			
Diabetes—% (*n*)	29% (11)	16.8% (105)	0.082
Arterial hypertension—% (*n*)	63.2% (24)	50.7% (318)	0.255
Dyslipidemia—% (*n*)	26.3% (10)	20.1% (126)	0.462
Family history of cardiovascular disease—% (*n*)	10.5% (4)	21.2% (133)	0.10
Smoking—% (*n*)	26.3% (10)	44.5% (279)	**0.015**
*Comorbidities*			
Coronary artery disease^1^—% (*n*)	39.5% (15)	14% (88)	** < 0.001**
Stroke—% (*n*)	13.2% (5)	4.5% (28)	**0.023**
Peripheral artery disease—% (*n*)	2.6% (1)	2.9% (18)	0.891
COPD—% (*n*)	13.2% (5)	8.1% (51)	0.334
Chronic kidney disease—% (*n*)	44.7% (17)	13.7% (86)	** < 0.001**
*Left ventricular structure/function*			
LV ejection fraction [%]	50 (40/55)	30 (20/40)	** < 0.001**
LV end-diastolic pressure (mmHg)	21 (17/25)	18 (13/25)	0.426
IVSED (cm)	1.6 (1.4/2.1)	1.1 (1/1.5)	**0.002**
Troponin I [pg/ml]	104 (45/239)	21 (8/59)	0.647
BNP [pg/ml]	456 (247/836)	408 (100/1,185)	0.352
*Signs and symptoms at presentation*			
NYHA I/II—% (*n*)	50% (19)	58.2% (365)	0.264
NYHA III/IV—% (*n*)	50% (19)	41.8% (262)	
Angina pectoris—% (*n*)	13.2% (5)	25.7% (161)	0.06
Oedema—% (*n*)	29% (11)	20.9% (131)	0.331

A p-value < 0.05 marked in bold letters suggests statistical significance

Data are presented as relative and absolute frequencies of subjects for binary variables and median with 1st and 3rd quartile for continuous traits

*BMI* body mass index, *COPD* chronic obstructive pulmonary disease, *BNP* brain natriuretic peptide, *LV* left ventricular, *NYHA* New York Heart Association, *IVSED* interventricular septal thickness end-diastolic

^1^No history of myocardial infarction, and no relevant coronary stenosis revealed by invasive coronary angiography

**Fig. 3:**
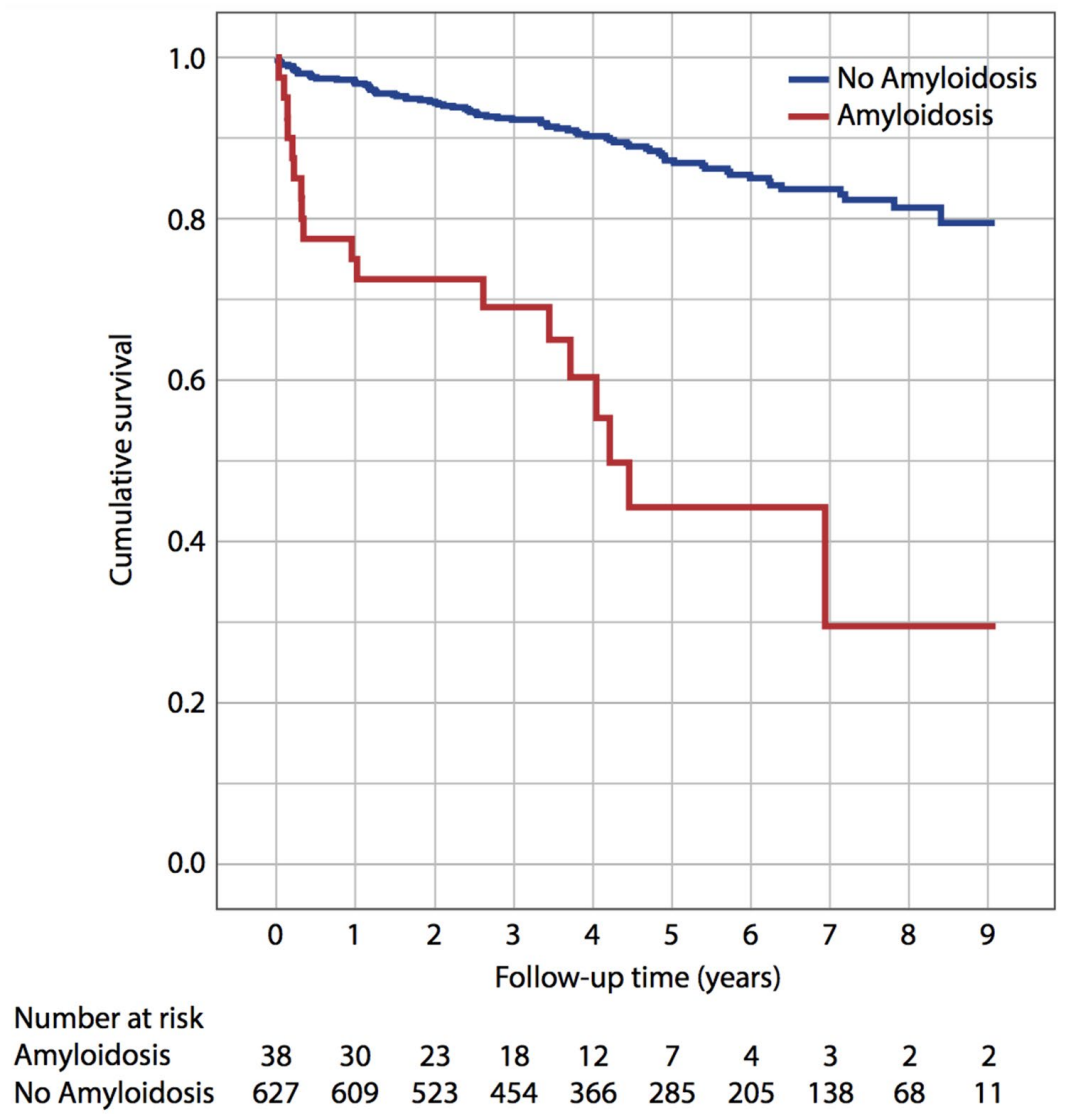
Long-term survival stratified for presence of cardiac amyloidosis. Kaplan–Meier curves indicating cumulative survival are displayed for patients with cardiac amyloidosis and those without cardiac amyloidosis

With respect to outcome, 1- and 5-year mortality rates were substantially higher in patients with CA compared to patients without CA (CA: 23.7% and 52.6% vs. no CA: 3.1% and 10.4%) as outlined in Fig. 3. The annualized event rates were 9 events/1 year and 20 events/5 years for patients with CA and 19 events/1 year and 65 events/5 years for patients without CA. Stratification of patients diagnosed with CA according to the subtype of amyloidosis (i.e. ATTRwt, ATTRv and AL-CA) revealed that 10 patients diagnosed with AL-CA, 5 patients diagnosed with ATTRwt and 2 patients diagnosed with ATTRv died during the observation period. Further stratification of patients without amyloidosis according to the most recent endomyocardial biopsy diagnoses (i.e. DCM, virus-negative cardiac inflammation, unspecific findings and others) revealed no statistically significant differences with respect to median survival. None of the patients diagnosed with giant cell myocarditis died during the observation period.

## Discussion

The main findings of the present analysis are: (i) EMB revealed cardiac inflammation in the majority of cases in our all-comer population of unexplained HF, mostly virus-negative; (ii) cardiac amyloidosis (including both, transthyretin amyloidosis as well as light-chain amyloidosis) was diagnosed in 5.8% of all cases of HF of unexplained etiology; and (iii) NYHA functional class III + IV, age and cardiac amyloidosis were independently associated with all-cause mortality in our study.

### Etiology of heart failure

The underlying causes of DCM-like HFrEF can be stratified in genetic and non-genetic. Since 160 patients (24.4%) showed unspecific EMB findings, genotyping might facilitate the diagnosis of the underlying cause of HF in these patients, although genetic testing is currently only recommended if a genetic cause is suspected following appropriate counselling [14]. Familial forms account for approximately 30–50% of all cases of DCM. In western populations, the genetic nature of cardiomyopathies with subsequent HF may easily be obscured considering the rather small size of contemporary families. Therefore, it is important to consider that also sporadic DCM cases can be due to (spontaneous) genetic mutations [15]. To date, variants in over 50 genes have been identified to be associated with DCM, with the evidence being strongest for titin (TTN), beta myosin heavy chain (MYH7), troponin2 (TNNT2) and the nuclear envelope gene lamin A/C (LMNA). All of these mutations accounted for the majority of detected mutations in our present analysis. Interestingly, Lota et al. recently provided evidence that DCM as well as arrhythmogenic cardiomyopathy-associated genetic variants are detectable in up to eight percent of patients with acute myocarditis. This finding highlights the potential role of genetic sequencing in patients presenting with acute myocarditis and supports the concept that at least some genotype-positive individuals may remain phenotypically silent until the occurrence of an environmental trigger [16]. We are not able to confirm these results in our study, since patients diagnosed with acute myocarditis did not undergo additional genotyping.

Once considered an orphan disease, recent studies have indicated that CA is quite common in selected patient populations and that it increases with age [17]. Most of these studies solely focused on populations with a high pre-test probability of CA. According to the results of these studies, prevalence of ATTRwt-CA was 6.3 to 33% in elderly patients with HFpEF [18–22], 9.3 to 11% in patients with HFmrEF/HFrEF [23, 24], 4 to 16% in elderly patients with aortic stenosis referred to hospital for surgical aortic valve replacement or transcatheter aortic valve replacement [25–29], 5 to 9% in elderly patients with a phenotype of hypertrophic cardiomyopathy [30, 31] and 3 to 10% in elderly patients undergoing carpal tunnel syndrome surgery [32–34].

Compared with the prevalence of CA reported in the above-mentioned meta-analysis [17], the prevalence detected in our study is lower (5.8%) across the whole spectrum of LVEF [18–20, 23, 24]. This is most likely attributable to the significantly younger population investigated in our study. 60.5% of our patients diagnosed with CA had ATTR-CA and 39.5% had AL-CA. This aligns with the published evidence: In six of the eight earlier studies on that topic, only ATTR-CA was detected, whereas two studies indicated that 20% [19] and 40% [22] of HFpEF patients diagnosed with CA had AL-CA, respectively.

### Outcome of patients with heart failure and predictors of all-cause mortality

Considering the phenotype of HF, survival rate is equivalent to or worse than many forms of cancer [35], and slightly higher in patients with HFrEF compared to HFpEF [36, 37]. Mortality rates significantly increase with age [38, 39] as indicated by 1-year and 5-year survival rates of 91.5% and 78.8% for people aged < 65 years and 83.3% and 49.5% for patients aged > 75 years [39]. Besides age, severity of HF as indicated by NYHA functional class is one of the most important clinical predictors of mortality in HF [40]. In our study, we recapitulated the importance of both age and NYHA class for outcomes. Body mass index was inversely associated with mortality, confirming the obesity paradox indicating worse outcome for individuals with low BMI [41] (see Fig. 2). The relatively young age of patients included in our analysis probably also accounts for the markedly lower prevalence of risk factors and comorbidities compared to other studies reporting on survival of patients with HF [36, 38]. This is of great importance as the burden of comorbidities in patients with HF increases with age and significantly impacts on survival [42].

With respect to non-genetic causes, chronic inflammatory responses represent a subtype of primary acquired DCM and EMB may help to detect possible underlying pathologies [43–45]. Both human herpesvirus 6 (HHV6) and erythroparvovirus B19 (B19V) account for most of virus-related inflammation, which is in line with the results of our present analysis. Development of subclinical myocarditis may be a sequel of chronic viral infection, leading to the progression from acute myocarditis to DCM. Kühl et al. reported that in individuals with a DCM-phenotype and increased pre-test probability of a history of viral myocarditis, viral genomes could be detected in endomyocardial biopsies in almost 67% of the patients [46]. The fact that 45.8% of the present study sample were diagnosed with cardiac inflammation emphasizes the involvement of immune responses in patients with non-ischemic HF. Spontaneous resolution of acute myocarditis associated with a substantial improvement in left ventricular ejection fraction and HF can be expected in up to 60% of patients. Data regarding the development of DCM following acute myocarditis are inconsistent. Prevalence estimates range from 14 to 52% [47, 48], making an inevitable sequence of events from myocarditis to inflammatory cardiomyopathy to DCM debatable.

In our study, perforin-positive cardiac inflammation was associated with worse outcome in a multivariate analysis adjusted for sex and age, recapitulating earlier findings by Escher et al. [49] Evidence of any type of cardiac inflammation in EMB, regardless of severity, was not associated with all-cause mortality in our population which was not preselected for high pretest probability of (post)myocarditis. Immunosuppressive therapy of virus-negative inflammatory cardiomyopathy has been investigated in the TIMIC study suggesting positive effects only in selected patients [50], a concept that is supported by the results of our study.

Interestingly, patients with CA showed high 1- and 5-year mortality rates, and evidence of CA was independently associated with mortality in our population, even after multiple adjustments for possible confounders. As cardiac involvement has a major prognostic impact in amyloidosis, current staging systems incorporate the extent of cardiac involvement as measured by serum markers in both AL-CA [51–53] and ATTR-CA [54]. Contemporary treatment options have significantly improved survival of patients not only with AL amyloidosis [55] but also with ATTR amyloidosis [56]. Survival of patients with ATTR-CA is significantly better in patients with early-stage ATTR-CA (> 100 months) compared to more advanced stages [57]. An early diagnosis is, therefore, mandatory in both AL-CA and ATTR-CA, a concept that is further supported by our study.

### Diagnostic considerations

According to current guidelines, the diagnosis of HF requires the presence of symptoms and/or signs of HF as well as objective evidence of cardiac dysfunction [3]. The proposed diagnostic algorithm includes an evaluation of risk factors, symptoms and signs of HF, electrocardiogram, measurement of natriuretic peptides (i.e. NT-proBNP or BNP), as well as echocardiographic examination to stratify for HFrEF, HFmrEF and HFpEF. Further imaging modalities like cardiac magnetic resonance (CMR) as well as invasive testing (i.e. coronary angiography and endomyocardial biopsy) should only be considered under appropriate conditions. For example, invasive coronary angiography is solely recommended in patients with angina despite pharmacological therapy or symptomatic ventricular arrhythmias (class I, level of evidence B) and endomyocardial biopsy should be considered in patients with rapidly progressive HF despite standard therapy and a high probability of a specific diagnosis (class IIa, level of evidence C). In our study, indication to perform EMB was based on the EMB guidelines that date back to 2007 [4]. Given the potential impact of EMB on risk stratification and disease management of non-ischemic and non-valvular HF, the results of our study call for new prospective trials to test the usefulness of a holistic work-up of HF including EMB to increase the level of evidence.

## Conclusion

The results of the present study demonstrate that incidental finding of cardiac amyloidosis represents a relevant differential diagnosis in an all-comer population with new onset of non-ischemic and non-valvular heart failure, being the underlying cause of HF in 5.8%. The relatively low 1- and 5-year mortality rates documented in the current study are probably attributable to the relatively young patient population, the low prevalence of comorbidities compared with other HF studies, and the exclusion of patients with ischemic heart failure. As CA was independently associated with excess mortality, our study underscores the need to improve screening methods that allow the diagnosis of CA at a very early stage, especially in light of the recently established causal/specific therapeutic options, that have proven to improve outcomes of CA.

## Limitations

The limitations of our retrospective monocentric study include lack of extended clinical data for all of the patients covered in this study and potential selection bias. Patients included in the present analysis were mainly of Caucasian ethnicity, which may limit the applicability of our findings to other ethnic groups. Furthermore, we were not able to provide information about causes of death and on recurrent heart failure related hospitalizations. The provided prevalence in the present study should also be interpreted with caution considering the retrospective study design, and HF practitioners should be mindful to apply our findings to their daily practice. Instead, our study suggests that prospective trials are needed to test the added value of a systematic and holistic work-up of HF of unknown etiology to improve diagnosis and management of HF.

### Supplementary Information

Below is the link to the electronic supplementary material.

Supplementary file 1 (DOCX 131 KB)

## Data Availability

The data and methods used to conduct the research in this study will be made available from the corresponding author on reasonable request.

## Abbreviations

AL: Immunoglobulin light chain amyloidosis
ATTRh: Hereditary transthyretin amyloidosis
ATTRwt: Wild-type transthyretin amyloidosis
CA: Cardiac amyloidosis
CAD: Coronary artery disease
CKD: Chronic kidney disease
COPD: Chronic obstructive pulmonary disease
CVRF: Cardiovascular risk factors
EMB: Endomyocardial biopsy
HF: Heart failure
HFmrEF: Heart failure with mildly reduced ejection fraction
HFpEF: Heart failure with preserved ejection fraction
HFrEF: Heart failure with reduced ejection fraction
HR: Hazard ratio
LV: Left ventricular
LVEF: Left ventricular ejection fraction
NYHA: New York Heart Association
