# Supplementary material for: Etiologies and predictors of mortality in an all-comer population of patients with non-ischemic heart failure
Source: Clin Res Cardiol. 2024 Jan 15;113(5):737–49. doi: 10.1007/s00392-023-02354-6 (PMC11026225; doi:10.1007/s00392-023-02354-6)
Supplement: Supplementary file 1 — (DOCX 131 KB) [file 392_2023_2354_MOESM1_ESM.docx]

**SUPPLEMENTARY APPENDIX**

**Etiologies and predictors of mortality in an all-comer population of patients with non-ischemic heart failure**

**Göbel S^1,2^, Braun AS^1^, Hahad O^1^, von Henning U^1^, Brandt M^1,2,3^, Keller K^1,2,3^, Gaida MM ^4,5^, Gori T^1,2^, Schultheiss HP^6^, Escher F ^7,8^, Münzel T^1,2^, Wenzel P^1,2,3^**

^1^ Cardiology I – Dept. of Cardiology, University Medical Center Mainz (Johannes Gutenberg

University Mainz), Mainz, Germany

^2^ German Center for Cardiovascular Research (DZHK), Partner Site Rhine Main, Mainz,

Germany

^3^ Center for Thrombosis and Hemostasis (CTH), University Medical Center Mainz (Johannes

Gutenberg-University Mainz), Mainz, Germany

^4^ Institute of Pathology, University Medical Center Mainz (Johannes Gutenberg University

Mainz), Mainz, Germany

^5^ TRON, Translational Oncology at the University Medical Center Mainz, Mainz, Germany

^6^ Institute of Cardiac Diagnostics and Therapy (IKDT), Berlin, Germany

^7^ Deutsches Herzzentrum der Charité, Department of Cardiology, Angiology and Intensice

Care Medicine, Campus Virchow Klinikum Berlin, Germany

^8^ German Center for Cardiovascular Research (DZHK), Partner Site Berlin, Berlin,

Germany

**Address for correspondence:**

Prof. Philip Wenzel, MD, FESC

Cardiology I – Dept. of Cardiology; University Medical Center Mainz of the Johannes Gutenberg-University Mainz

Langenbeckstr. 1; 55131 Mainz; Germany.

Telephone: 0049-6131-17-5462;

Telefax: 0049-6131-17-8461;

E-Mail: wenzelp@uni-mainz.de

ORCID: 0000-0002-5397-2781

**CONTENTS**

- **Echocardiography**
- **Endomyocardial biopsy**
- **Tissue processing, polymerase chain reaction, histology and immunohistochemistry**
- **Genotyping**
- **Supplemental Table 1**

Genotyping stratified for EMB based diagnosis

- **Supplemental Table 2**

Genotyping stratified for phenotype of heart failure

- **Supplemental Figure 1**

Markers of inflammation and all-cause mortality

**Echocardiography**

Left ventricular ejection fraction (LVEF) was assessed by biplane Simpson’s method in the apical four and two chamber view. Mitral inflow velocity pattern was recorded from the apical four-chamber view with the pulsed waved Doppler sample volume positioned at the tips of the mitral valve leaflets during diastole in expiration. Peak early (E-wave) and late (A-wave) diastolic filling velocities were measured and their ratio (E/A) calculated. The lateral and septal mitral annular early diastolic velocity (E’) were measured by spectral tissue Doppler imaging and the E/E’ ratio determined. For the present analysis, HF was categorized in three classes based on left ventricular ejection fraction (LVEF ≥ 50% [HFpEF]; LVEF 41–49% [HF with mid-range reduced LVEF=HFmrEF] and LVEF<40% [HFrEF) according to the recently published guidelines for the diagnosis and treatment of acute and chronic heart failure by the European Society of Cardiology (ESC)^1^.

**Endomyocardial biopsy**

The 7.5F Eaucath system (Asahi Intecc) was originally designed as a sheathless guiding catheter for the use in transradial coronary interventions and used for transradial LV- EMB. For RV-EMB, we used a 9F FastCath system. For transfemoral LV-EMB we used either an 8F MP GC system or a 7.5F Eaucath system (Asahi Intecc; sheathless).

Patients undergoing LV-EMB through the femoral access only received 5,000 IU intraarterial, whereas patients undergoing RV-EMB did not routinely receive heparin nor nitroglycerin. No heparin was administered intra-procedurally in both patients undergoing LV-EMB through the radial artery and in patients undergoing LV-EMB through the femoral artery if they were treated with vitamin-K antagonists and laboratory results documented an INR > 2.5. In case of elective EMB procedures and concomitant DOAC therapy, the drug was paused 24 hours before the procedures. Use of heparin was monitored intra procedurally by measuring the activated clotting time (target ACT: >250s). In all patients irrespective of the access site, the same type of biopsy forceps (Medwork bioptom, 180cm, 1.8mm, Cat.-No. BIO-C4-18-180) was used. The technical aspects of EMB through the radial artery have been described in detail previously^2,3^.

**Tissue processing, polymerase chain reaction, histology and immunohistochemistry**

Immediately after sampling the biopsies, the specimens were stabilized in formalin or a solution to preserve ribonucleic acid (RNA) integrity (RNAlater, Thermo Fisher Scientific, Waltham, MA, USA), respectively, and sent for further examination to a specialized CAP-accredited laboratory [Institute for Cardiac Diagnostics and Therapy (IKDT), Berlin, Germany]. As part of a routinely performed work up in case of unclear heart failure, histological and immunohistochemical examinations as well as viral genome detection were conducted. Histologic examinations were performed on formalin-fixed paraffin-embedded specimens stained with Hematoxylin and Eosin (H&E), Azan, Elastika-van-Gieson (EvG) and periodic acid-Schiff (PAS) reagents according to standard procedures. Active myocarditis was diagnosed according to the histopathological Dallas criteria^4^. Immunohistochemical evaluations were performed on RNAlater-fixed cryo-embedded EMBs. Briefly, specimens were embedded in Shandon™ Cryomatrix (Thermo Fisher Scientific, Waltham, MA, USA), cut serially into cryosections of 5 µm thickness and placed on 10% poly‐L‐lysine‐precoated slides. Using specific antibodies inflammatory processes were detected by identifying immune cell infiltration and expression of cell adhesion molecules. According to the European Society of Cardiology (ESC) statement^5^, myocardial inflammation was diagnosed by the detection of ≥ 14 leukocytes per mm², with the presence of ≥ 7 CD3+ T-lymphocytes per mm² (Dako; dilution 1:700). In addition, ≥ 14 LFA-1+ lymphocytes per mm² (ImmunoTools; dilution 1:250), ≥ 35 MAC-1+ macrophages per mm² (ImmunoTools; dilution 1:500), ≥ 40 CD45R0+ memory T cells per mm² (Dako, dilution 1:600), and ≥ 2.9 perforin+ cytotoxic cells per mm² (BD Bioscience; dilution 1:150) were considered pathologic. Endothelial activation was measured by the expression of adhesion molecules HLA-DR (Antikoerper-online; dilution 1:100, threshold ≥ 2 area%) and ICAM-1 (ImmunoTools, dilution 1:800, threshold ≥ 2 area%). All immunohistochemical markers were quantified by digital image analysis as previously described^6^.

Diagnosis of relevant myocardial virus infections was performed by polymerase chain reaction (PCR) detecting the genomic sequences of viruses that most commonly cause myocarditis. This included: parvovirus B19, enterovirus, adenovirus, human cytomegalovirus, herpes simplex virus, Epstein-Barr virus, human herpesvirus 6, and influenza A and B viruses. Virus load was calculated via quantitative PCR methods. Transcriptional activity of parvovirus B19 was determined in as described before^7,8^.

The definitive diagnosis of cardiac amyloidosis was obtained through tissue biopsy stained with Congo red which shows pathognomonic green birefringence of amyloid deposits when viewed under polarized light. Further typing of amyloidosis was performed immunohistochemically using amyloid type specific antibodies (‘amY-kit’ from www.amYmed.net), which are directed against the amyloidotic conformational epitopes of amyloid^9^.

**Genotyping**

Using NGS (Next-Generation Sequencing), the coding exons of the relevant genes were analyzed on the basis of the genomic DNA. Here, the corresponding target regions were enriched with the corresponding exon-intron transitions using Twist Bioscience technology (Twist Clinical Exome). Sequencing (paired-end run) was performed using Illumina technology on a NextSeq550/2000 instrument. A minimum of 15-fold sequencing depth (coverage) was achieved for the gene segments of interest for diagnostic significance. The quality of the NGS diagnostics performed is in accordance with the ESHG stated^10^. Type A: The laboratory guarantees > 99% coverage of target sequences, any gaps are completely filled by sanger sequencing; Type B: The laboratory describes which regions are > 99% covered, partially fills any gaps by sanger sequencing, and indicates uncovered regions; Type C: This test is based on the quality of NGS sequencing alone, no additional sanger sequencing is provided. Analysis of NGS data was performed using varvis® bioinformatics analysis software, version 1.20 (Limbus Medical Technologies GmbH, Rostock, Germany). Identified variants and indels were matched against various external and internal databases and filtered based on their allele frequency, with only rare variants with MAF below 1% (excluding variants clearly described as pathogenic with higher MAF) considered for further analysis. The obtained variants were matched against the patient's clinical data known to us. Variants were classified according to ACMG/AMP guidelines^11^ and ACGS taking into account the current literature and database situation (internal/external mutation and allele frequency databases, published clinical and functional studies, family history/segregation data) as well as clinical data/laboratory parameters and bioinformatics prediction programs. Primarily pathogenic and likely pathogenic variants are considered for reporting. Variants of uncertain significance (VUS) may also be reported depending on clinical symptoms if data are convincing. We also reserve the right not to report (probably) pathogenic heterozygous variants in clearly autosomal recessive genes that alone do not explain the phenotype. Variants classified as (probable) normative variants without clinical relevance [(probable) benign] are not reported. A change in the classification of variants at a later stage due to changing data cannot be excluded. Reported deviations from the human gene-specific reference sequence (according to Genome Reference Consortium Human Build 37, GRCh37/hg19) were evaluated using an internal quality system. Major findings reported variants that did not pass the quality assessment threshold were validated by Sanger sequencing. Validation of variants listed as secondary findings was not performed as a rule. Sample identity was ensured by internal quality management procedures. Based on the NGS data, the detection of copy number variations) is possible in principle. Nevertheless, it cannot be excluded that the detection of structural changes (e.g. deletions, duplications) in certain target regions or of single exon deletions/duplications is not unequivocal. In known complex genomic regions, matching by MLPA (multiplex ligation-dependent probe amplification) or alternative methods is usually performed, if necessary, to validate positive results.

**Supplemental Table S1. Genotyping stratified for EMB based diagnosis**

| **Characteristics** | **Study sample**  **N = 655** | **Genotyping (yes)**  **N = 113** | **Genotyping (no)**  **N = 542** |
| --- | --- | --- | --- |
| Unspecific finding - % (n) | 143 | 18.2 % (26) | 81.8% (117) |
| Dilative Cardiomyopathy - % (n) | 78 | 25.6% (20) | 74.4% (58) |
| Hypertrophic cardiomyopathy - % (n) | 15 | 40% (6) | 60% (9) |
| Hypertensive heart disease - % (n) | 4 | 0% (0) | 100% (4) |
| ARVD - % (n) | 2 | 50% (1) | 50% (1) |
| Amyloidosis - % (n) | 38 | 60.5% (23) | 39.5% (15) |
| Sarcoidosis - % (n) | 5 | 0% (0) | 100% (5) |
| Post myocarditis - % (n) | 65 | 4.6% (3) | 95.4% (62) |
| Active myocarditis - % (n) | 6 | 0% (0) | 100% (6) |
| Erythroparvovirus without inflammation  - % (n) | 31 | 12.9% (4) | 87.1% (27) |
| Erythroparvovirus with inflammation –  % (n) | 9 | 0% (0) | 100% (9) |
| Coxsackie without inflammation - % (n) | 4 | 0% (0) | 100% (4) |
| Coxsackie with inflammation - % (n) | 1 | 0% (0) | 100% (1) |
| Erythroparvovirus/HHV reactivation  without  Inflammation - % (n) | 14 | 7.1% (1) | 92.9% (13) |
| Giant cell myocarditis - % (n) | 6 | 0% (0) | 100% (6) |
| Toxic cardiomyopathy - % (n) | 4 | 50% (2) | 50% (2) |
| Virus negative inflammatory CM - % (n) | 215 | 11.6% (25) | 88.4% (190) |

Data are presented as relative and absolute frequencies of subjects for binary variables. ARVD, arrythmogenic right ventricular dysplasia

**Supplemental Table S2. Genotyping stratified for phenotypes of heart failure**

| **Mutations** | **HFrEF**  **N = 489** | **HFmrEF**  **N = 52** | **HFpEF**  **N = 114** |
| --- | --- | --- | --- |
| BAG3 - % (n) | 0.2% (1) | 0% (0) | 0% (0) |
| VUS - % (n) | 0.2% (1) | 1.9% (1) | 0.9% (1) |
| FLNC - % (n) | 0.2% (1) | 0% (0) | 0.9% (1) |
| FLNC / JPH2 - % (n) | 0% (0) | 0% (0) | 0.9%(1) |
| LMNA - % (n) | 1% (5) | 0% (0) | 0% (0) |
| MYBPC3 - % (n) | 0.6% (3) | 0% (0) | 0.9% (1) |
| MYH7 - % (n) | 0.4% (2) | 0% (0) | 0% (0) |
| MYPN - % (n) | 0.2% (1) | 0% (0) | 0% (0) |
| MYOM1 - % (n) | 0.2% (1) | 0% (0) | 0% (0) |
| OBSL-1 - % (n) | 0% (0) | 0% (0) | 0.9% (1) |
| SCN10A - % (n) | 0.2% (1) | 0% (0) | 0% (0) |
| LAMA4 - % (n) | 0.2% (1) | 0% (0) | 0% (0) |
| TTN - % (n) | 1.2% (6) | 1.9% (1) | 0% (0) |
| TTN / JUP - % (n) | 0.2% (1) | 0% ()0 | 0% ()0 |
| TTN / MYBPC3 - % (n) | 0.2% (1) | 0% (0) | 0% (0) |
| CSPR3 / GLA - % (n) | 0% (0) | 0% (0) | 0.9% (1) |
| ***Amyloidosis specific mutation*** |  |  |  |
| Val 30 Met - % (n) | 0% (0) | 0% (0) | 2.7% (3) |
| pIIe127 Val - % (n) | 0% (0) | 1.9% (1) | 0% (0) |
| pIIe18 Leu - % (n) | 0% (0) | 0% (0) | 0.9% (1) |
| pVal142 IIe - % (n) | 0.2% (1) | 0% (0) | 0% (0) |

Data are presented as relative and absolute frequencies of subjects for binary variables. HFrEF indicates

heart failure with reduced ejection fraction; HFmrEF, heart failure with mildly reduced ejection fraction;

HFpEF, heart failure with preserved ejection fraction.

**Supplemental Figure 1. Markers of inflammation and all-cause mortality**

**
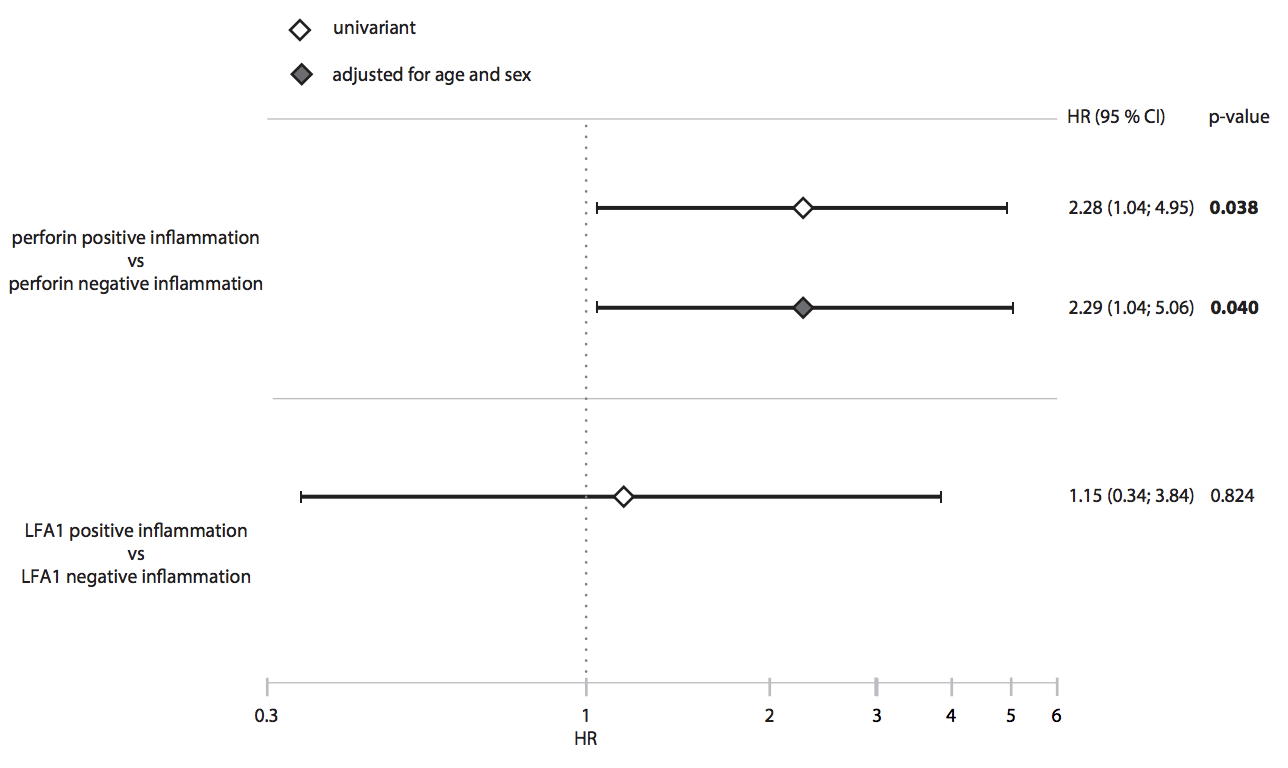
**

HR indicates hazard ratio; CI, confidence interval; LFA1, lymphocyte function-associated antigen 1.

**References**

1. McDonagh TA, Metra M, Adamo M, Gardner RS, Baumbach A, Bohm M, Burri H, Butler J, Celutkiene J, Chioncel O*, et al.* 2021 ESC Guidelines for the diagnosis and treatment of acute and chronic heart failure. *Eur Heart J* 2021;**42**:3599-3726. doi: 10.1093/eurheartj/ehab368

2. Schulz E, Jabs A, Gori T, Hink U, Sotiriou E, Tschope C, Schultheiss HP, Munzel T, Wenzel P. Feasibility and safety of left ventricular endomyocardial biopsy via transradial access: Technique and initial experience. *Catheter Cardiovasc Interv* 2015;**86**:761-765. doi: 10.1002/ccd.25834

3. Gobel S, Schwuchow-Thonke S, Jansen T, Karbach S, Emrich T, Gori T, Knies F, Schulz E, Munzel T, Keller K*, et al.* Safety of transradial and transfemoral left ventricular compared with transfemoral right ventricular endomyocardial biopsy. *ESC Heart Fail* 2020. doi: 10.1002/ehf2.13006

4. Aretz HT. Myocarditis: the Dallas criteria. *Hum Pathol* 1987;**18**:619-624. doi: 10.1016/s0046-8177(87)80363-5

5. Caforio AL, Pankuweit S, Arbustini E, Basso C, Gimeno-Blanes J, Felix SB, Fu M, Helio T, Heymans S, Jahns R*, et al.* Current state of knowledge on aetiology, diagnosis, management, and therapy of myocarditis: a position statement of the European Society of Cardiology Working Group on Myocardial and Pericardial Diseases. *Eur Heart J* 2013;**34**:2636-2648, 2648a-2648d. doi: 10.1093/eurheartj/eht210

6. Escher F, Kuhl U, Lassner D, Stroux A, Gross U, Westermann D, Pieske B, Poller W, Schultheiss HP. High Perforin-Positive Cardiac Cell Infiltration and Male Sex Predict Adverse Long-Term Mortality in Patients With Inflammatory Cardiomyopathy. *J Am Heart Assoc* 2017;**6**. doi: 10.1161/JAHA.116.005352

7. Pietsch H, Escher F, Aleshcheva G, Lassner D, Bock CT, Schultheiss HP. Detection of parvovirus mRNAs as markers for viral activity in endomyocardial biopsy-based diagnosis of patients with unexplained heart failure. *Sci Rep* 2020;**10**:22354. doi: 10.1038/s41598-020-78597-4

8. Escher F, Aleshcheva G, Pietsch H, Baumeier C, Gross UM, Schrage BN, Westermann D, Bock CT, Schultheiss HP. Transcriptional Active Parvovirus B19 Infection Predicts Adverse Long-Term Outcome in Patients with Non-Ischemic Cardiomyopathy. *Biomedicines* 2021;**9**. doi: 10.3390/biomedicines9121898

9. Linke RP. On typing amyloidosis using immunohistochemistry. Detailled illustrations, review and a note on mass spectrometry. *Prog Histochem Cytochem* 2012;**47**:61-132. doi: 10.1016/j.proghi.2012.03.001

10. Matthijs G, Souche E, Alders M, Corveleyn A, Eck S, Feenstra I, Race V, Sistermans E, Sturm M, Weiss M*, et al.* Guidelines for diagnostic next-generation sequencing. *Eur J Hum Genet* 2016;**24**:1515. doi: 10.1038/ejhg.2016.63

11. Richards S, Aziz N, Bale S, Bick D, Das S, Gastier-Foster J, Grody WW, Hegde M, Lyon E, Spector E*, et al.* Standards and guidelines for the interpretation of sequence variants: a joint consensus recommendation of the American College of Medical Genetics and Genomics and the Association for Molecular Pathology. *Genet Med* 2015;**17**:405-424. doi: 10.1038/gim.2015.30
